# A machine learning strategy to mitigate the inappropriateness of procalcitonin request in clinical practice

**DOI:** 10.1016/j.heliyon.2024.e26556

**Published:** 2024-02-17

**Authors:** Luisa Agnello, Matteo Vidali, Anna Maria Ciaccio, Bruna Lo Sasso, Alessandro Iacona, Giuseppe Biundo, Concetta Scazzone, Caterina Maria Gambino, Marcello Ciaccio

**Affiliations:** a Institute of Clinical Biochemistry, Clinical Molecular Medicine and Clinical Laboratory Medicine, Department of Biomedicine, Neurosciences and Advanced Diagnostics, University of Palermo, 90127, Palermo, Italy; b Foundation IRCCS Ca’ Granda Ospedale Maggiore Policlinico, 20122, Milan, Italy; c Internal Medicine and Medical Specialties “G. D'Alessandro”, Department of Health Promotion, Maternal and Infant Care, University of Palermo, 90127, Palermo, Italy; d Department of Laboratory Medicine, AOUP “P. Giaccone”, Palermo, Italy

**Keywords:** Laboratory medicine, Sepsis, Procalcitonin, MDW, CRP, Artificial intelligence, CBC

## Abstract

**Aim:**

The aim of this study was to develop machine learning (ML) models to mitigate the inappropriate request of Procalcitonin (PCT) in clinical wards.

**Material and methods:**

We built six different ML models based on both demographical data, i.e., sex and age, and laboratory parameters, i.e., cell blood count (CBC) parameters, inclusive of monocyte distribution width (MDW), and C-reactive protein (CRP). The dataset included 1667 PCT measurements of different patients. Based on a PCT cut-off of 0.50 ng/mL, we found 1090 negative (65.4%) and 577 positive (34.6%) results. We performed a 70:15:15 train:validation:test splitting based on the outcome.

**Results:**

Random Forest, Support Vector Machine and eXtreme Gradient Boosting showed optimal performances for predicting PCT positivity, with an area under the curve ranging from 0.88 to 0.89.

**Conclusions:**

The ML models developed could represent a useful tool to predict PCT positivity, avoiding unusefulness PCT requests. ML models are based on laboratory tests commonly ordered together with PCT but have the great advantage to be easy to measure and low-cost.

## 1 Introduction

Procalcitonin (PCT) is a 116-amino acid peptide synthesized by the calcitonin-1 gene (CALC-1) localized on chromosome 11. Under physiological conditions, the product of CALC-1 is the prePCT, which is first processed into PCT and then in calcitonin within thyroid C-cells [1]. Under pathological conditions, PCT synthesis is activated in all parenchymal tissues and its levels can increase to 100 to 1000-fold [2]. Specifically, PCT production is stimulated by endotoxins and pro-inflammatory cytokines, such as interleukin-6, tumor necrosis factor-α, and interleukin-1β, whose levels increase upon bacterial infection. In contrast, viral infection down-regulates PCT synthesis by interferon-gamma.

Generally, PCT levels are very low in healthy individuals (<0.1 ng/mL). PCT synthesis starts within 3–4 h following an infection, reaches the peaks at 6–12 h, and its levels correlate with the severity of the infection. It has a half-life of about 24 h. Beyond bacterial infection, several clinical conditions may induce circulating PCT increase, such as trauma, severe burns, major surgery, cardiogenic shock, small cell lung carcinoma, and chronic kidney disease [3]. Additionally, some drugs, such as alemtuzumab or OKT3, could stimulate cytokine production leading to PCT synthesis. Finally, PCT is not routinely elevated in immunocompromised patients or with atypical bacterial infections [4].

In clinical practice, PCT is a biomarker widely ordered in different clinical wards, from the Emergency Department (ED) to the Intensive Care Unit (ICU) [[5], [6], [7], [8]]. PCT evaluation aids in guiding antibiotic therapy in critically ill patients [[9], [10], [11]]. However, PCT is an overhyped test, as defined by Paudel et al., leading to its inappropriate overuse [12]. Several strategies have been employed to reduce the rate of inappropriate test ordering to improve outcomes and reduce healthcare costs. These last represent two key factors in the Medicine of the Third Millennium, defined as patient-centered and focused on reducing healthcare costs.

The aim of this study was to develop machine learning (ML) models based on inexpensive and easy-to-measure laboratory parameters to reduce PCT overutilization.

## 2 Material and Methods

### 2.1 Study design

**Fig. 1:**
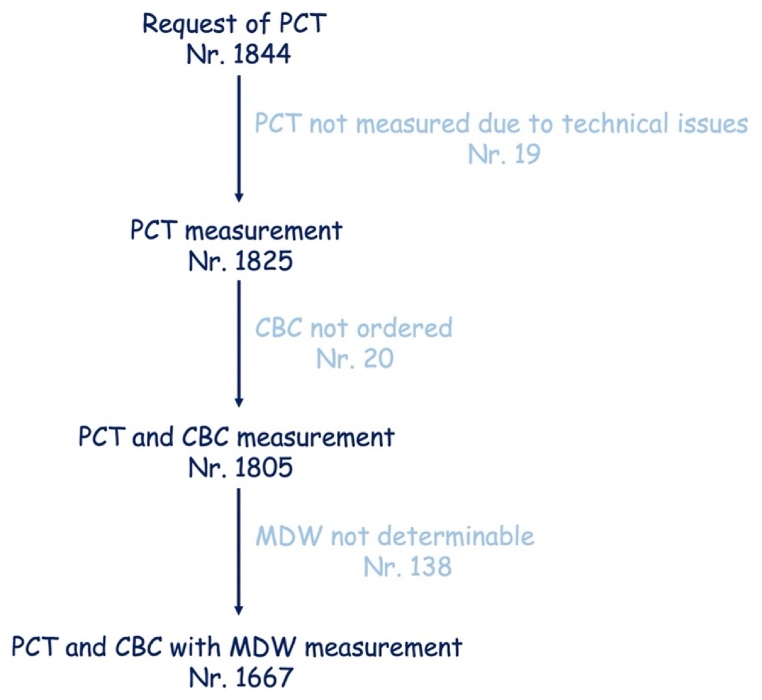
Flow-chart of the data included in the study.

In this observational retrospective monocenter study, we considered all PCT orders from January 01 to February 21, 2023, at the Department of Laboratory Medicine, University Hospital “P. Giaccone”, Palermo, Italy. We included in the study all PCT orders associated with the request of C-reactive protein (CRP) and monocyte distribution width (MDW). We excluded the orders lacking CRP and/or MDW. Orders were not selected according to age, sex, clinical ward, cause of the hospitalization, treatment, or other. The flowchart of the data included in the study is depicted in Fig. 1.

### 2.2 Laboratory analysis

Serum PCT was measured by an enzyme-linked fluorescence assay (miniVIDAS® BRAHMS PCT assay; Biomerieux, Lyon, France), according to the manufacturer. Serum CRP was measured by the latex enhanced immunoturbidimetric assay on a Cobas c702 analyzer (Roche Diagnostics), according to the manufacturer. MDW was calculated by the UniCel DxH 900 hematology analyzer (Beckman.

Coulter, Inc., Brea, California) in whole-blood sample collected in K3-EDTA tube.

### 2.3 Statistical analysis

#### 2.3.1 Statistical software

Statistical analyses were performed by R Language v.4.2.3 (R Foundation for Statistical Computing, Vienna, Austria) and RStudio v.2023.03.0 + 386, with additional packages haven, dplyr, skimr, Amelia, caret, Boruta, and doParallel.

#### 2.3.2 Dataset

The dataset included 1667 observations. We defined a PCT cut-off of 0.50 ng/mL. Based on such a cut-off, we found 1090 negative (65.4%) and 577 positive (34.6%) samples. Median (IQR) PCT values in the two groups were, respectively, 0.13 ng/mL (0.08–0.24) and 1.51 ng/mL (0.82–3.23).

We considered 21 variables: 1 outcome (PCT positivity) and 20 predictors (age, sex, white blood cells (WBC), neutrophils (NEU), lymphocytes (LYM), monocytes (MON), eosinophils (EOS), basophils (BAS), MDW, red blood cells (RBC), hemoglobin (Hb), hematocrit (HCT), mean corpuscular volume (MCV), mean corpuscular hemoglobin (MCH), mean corpuscular hemoglobin concentration (MCHC), red distribution width (RDW), platelets (PLT), platelet distribution width (PDW), mean platelet volume (MPV), and CRP). Continuous PCT values were transformed into binary variables (pos:PCT>0.5 ng/mL, neg:PCT≤0.5 ng/mL).

#### 2.3.3 Descriptive statistics

Normality distribution was assessed preliminarily by q-q plot and Shapiro–Wilk test. Quantitative variables were expressed by median and interquartile range (IQR), while qualitative variables by absolute or relative frequency.

#### 2.3.4 Train, validation, and test split

We performed a 70:15:15 train:validation:test splitting based on the outcome. The training set included 1167 patients (PCT pos: 405, 34.7%), while both the validation and the testing sets included 250 patients (PCT pos: 86, 34.4%).

#### 2.3.5 Missing data treatment

No missing data were found in the whole dataset.

#### 2.3.6 Feature selection

**Fig. 2:**
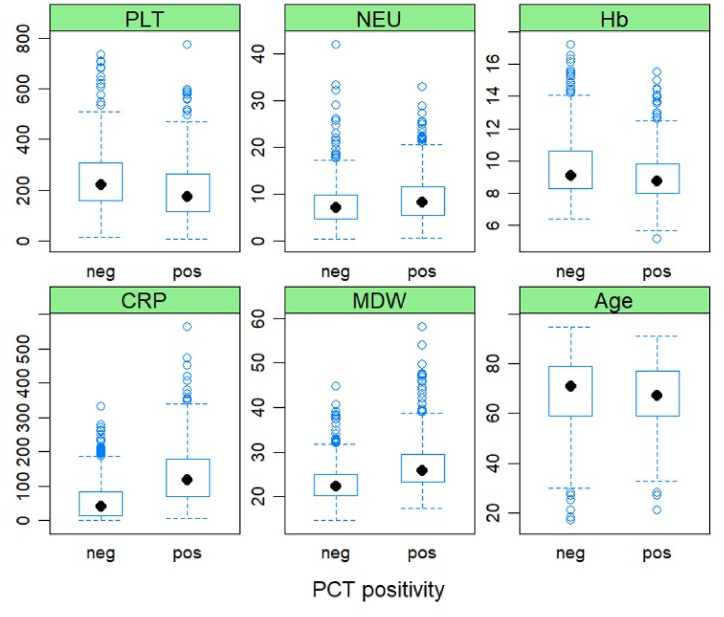
Boxplots for the distributions of six relevant features for predicting procalcitonin positivity, according to both recursive feature elimination and Boruta algorithms.

Unsupervised feature selection was performed by near-zero variance analysis and collinearity analysis. WBC, RBC, HCT, and MCH were removed due to high collinearity. For models available in the R caret packages, without built-in feature selection, supervised feature selection was performed by recursive feature elimination, using random forest and 10-fold cross-validation, and by the Boruta algorithm. Fig. 2 shows the distributions of six relevant features for predicting procalcitonin positivity, according to both recursive feature elimination and Boruta algorithms.

PLT: platelets, NEU: neutrophils, Hb: Hemoglobin, CRP: C-reactive protein, MDW: monocyte distribution width.

#### 2.3.7 Data preprocessing

Variables were normalized within the modeling train function of the R caret package.

#### 2.3.8 Algorithms and hyperparameters tuning

The following 6 classification algorithms were built: 1) Logistic Regression (LR) by the “glm” method (using forward approach by Akaike Information Criterion); 2) Naïve Bayes (NB) by the “nb” method (parameters: Laplace correction, distribution type, bandwidth adjustment), 3) Random Forest (RF) by the “rf” method (parameter: number of randomly selected predictors), 4) Support Vector Machines with Radial Kernel (SVMr) by the “svmRadial” method (parameters: sigma, cost), 5) eXtreme Gradient Boosting (XGB) by the “xgbTree” method (parameters: number of boosting iterations, max tree depth, shrinkage, minimum loss reduction, subsample ratio of columns, minimum sum of instance weight, subsample percentage), 6) k-nearest neighbors (KNN) by the “knn” method (parameter: number of nearest neighbors). Parameters were tuned to maximise AUC, using repeated 10-Fold Cross-Validation (repeats = 5) grid search.

#### 2.3.9 Model calibration and performance evaluation

After hyperparameters tuning, models were calibrated using the validation dataset and the final models were applied to the testing dataset. Model performances (accuracy, kappa, sensitivity, specificity, positive and negative predictive value) were calculated by a confusion matrix.

## 3 Results

In this study, we included 1667 data (M:F 56:44%). Demographic characteristics and results of biochemical tests are reported in Table 1. Median (IQR) levels of CRP and MDW were, respectively, 64.6 (25.1–119.0) mg/L and 23.5 (21.0–26.3) (Table 1). Up to 577 (34.6%) PCT measurements displayed levels>0.50 ng/mL (Table 1).

**Table 1:** Characteristics of the whole sample investigated.

Variable	Result (% or median, IQR)
Demographic	
**N**	1667
**Sex, M**	56%
**Age, years**	70 (60–78)
Biochemical	
**WBC, 10^9/L**	9.70 (7.00–13.10)
**NEU, 10^9/L**	7.50 (5.20–10.60)
**LYM, 10^9/L**	1.00 (0.60–1.50)
**MON, 10^9/L**	0.70 (0.40–1.00)
**Hemoglobin, g/L**	90 (82–104)
**RBC, 10^12/L**	3.11 (2.78–3.65)
**Ht, %**	26.5 (24.0–31.1)
**RDW, %**	15.7 (14.5–17.0)
**PLT, 10^9/L**	206 (142–293)
**CRP, mg/L**	64.6 (25.1–119.0)
**MDW**	23.5 (21.0–26.3)
**PCT** > **0.50 ng/mL**	35%

Patients with PCT>0.5 ng/mL (n = 577), with respect to patients with PCT≤0.5 ng/mL, displayed significantly higher WBC (p < 0.001), NEU (p < 0.001), MON (p = 0.025), CRP (p < 0.001), MDW (p < 0.001) and lower Hb (p < 0.001), RBC (p < 0.001), Ht (p < 0.001), RDW (p = 0.010) and PLT (p < 0.001) (Table 2, Fig. 2). Performances of the ML models, calculated on the test dataset, are reported in Table 3.

**Table 2:** Characteristics of the groups (PCT≤0.5 vs PCT>0.5 ng/mL). Continuous variables are expressed as median and interquartile range (IQR).

Variable	PCT≤0.5 ng/mL	PCT>0.5 ng/mL	p-value
Demographic			
**N**	1090	577	
**Sex, M**	57%	53%	0.133
**Age, years**	72 (60–80)	68 (60–77)	0.009
Biochemical			
**WBC, 10^9/L**	9.30 (6.80–12.30)	10.70 (7.50–14.90)	<0.001
**NEU, 10^9/L**	7.10 (5.00–10.00)	8.60 (5.65–11.90)	<0.001
**LYM, 10^9/L**	1.00 (0.60–1.40)	1.10 (0.60–1.50)	0.481
**MON, 10^9/L**	0.60 (0.40–0.90)	0.70 (0.40–1.10)	0.025
**Haemoglobin, g/L**	91 (83–107)	88 (80–100)	<0.001
**RBC, 10^12/L**	3.19 (2.83–3.73)	2.99 (2.67–3.51)	<0.001
**Ht, %**	26.8 (24.3–31.8)	25.9 (23.3–29.8)	<0.001
**RDW, %**	15.8 (14.6–17.1)	15.5 (14.3–16.7)	0.010
**PLT, 10^9/L**	221 (156–309)	176 (117–267)	<0.001
**CRP, mg/L**	42.9 (13.9–84.3)	115.0 (66.4–180.0)	<0.001
**MDW**	22.4 (20.4–25.0)	25.7 (23.1–29.2)	<0.001

**Table 3:** Performances of the ML models, calculated on the test dataset.

Model	Accuracy	kappa	Sensitivity	Specificity	PPV	NPV	AUC
LR	0.73	0.39	0.53	0.84	0.64	0.77	0.78
RF	0.82	0.58	0.69	0.88	0.76	0.84	0.89
NB	0.75	0.40	0.49	0.88	0.69	0.77	0.79
KNN	0.78	0.50	0.59	0.88	0.73	0.80	0.83
SVMr	0.81	0.57	0.65	0.90	0.77	0.83	0.89
XGB	0.82	0.57	0.64	0.91	0.79	0.83	0.88

**Fig. 3:**
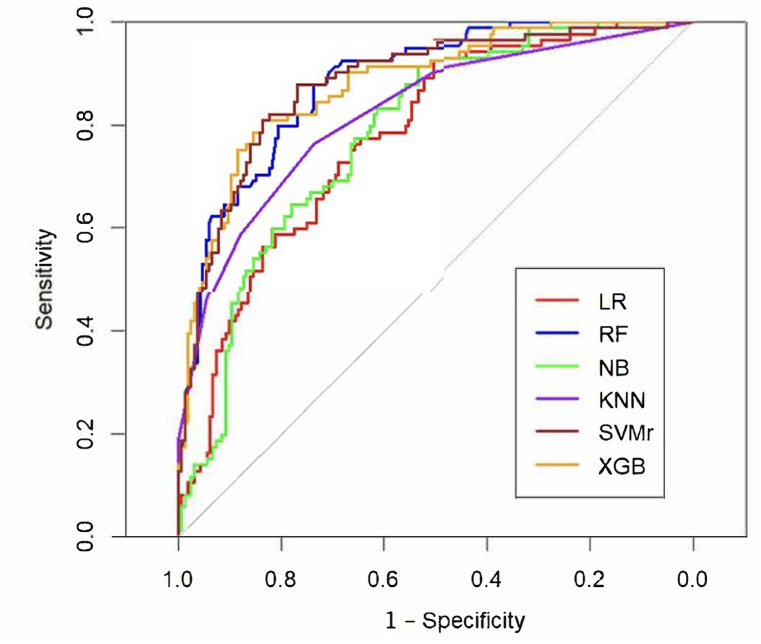
Receiver operating characteristic curves for the ML models evaluated.

Random Forest, Support Vector Machine, and eXtreme Gradient Boosting showed the highest performances in terms of accuracy, kappa, and sensitivity. The receiver operating characteristic (ROC) curves of the ML models evaluated are reported in Fig. 3, with RF, SVMr and XGB showing the highest AUCs, ranging from 0.88 to 0.89.

**Fig. 4:**
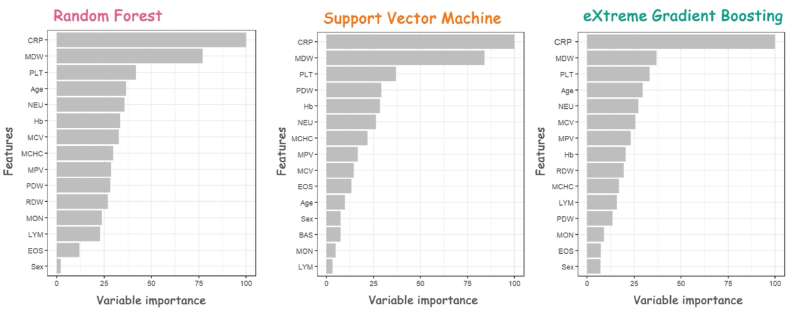
Variable importance scores for the Random Forest, Support Vector Machine, and eXtreme Gradient Boosting models.

As comparison, the AUCs of univariate models including only MDW or CRP, or of the multivariate model including their combination, were, respectively, 0.73 (95%CI 0.70–0.76), 0.78 (0.76–0.80) or 0.79 (95%CI 0.77–0.81). Variable importance scores for the three final models are shown in Fig. 4. For all three final ML models sex plays only a minor part and could be theoretically omitted without remarkably changing model performances (Fig. 4).

## 4 Discussion

PCT is a widely used biomarker in clinical practice. Since Food and Drug Administration cleared the use of PCT to aid antibiotic decision-making in hospitalized patients with sepsis and lower respiratory tract infections in 2017, its request has ballooned [13].

According to the literature evidence and the current guidelines, PCT is recommended in critically ill patients to guide antibiotic therapy de-escalation [14,15]. Nevertheless, most clinicians overestimate the value of PCT, ordering it for several reasons, leading to its inappropriate use. The main inappropriate reason for ordering PCT includes the diagnosis of sepsis and initiating antibiotic therapy, which are not supported by scientific data. Indeed, several Authors showed that PCT is not a reliable sepsis diagnostic tool due to poor sensitivity and the lack of a definite decisional cut-off [12]. The clinical usefulness of PCT in patients with suspected bacterial infection with a low risk of sepsis is low. Additionally, vast amounts of data indicate that PCT is more reliable in guiding the discontinuation than the initiation of antibiotics [12,16]. Finally, some limitations of PCT must be mentioned. Despite the good NPV for bacteremia, its PPV is not optimal, and several clinical conditions may lead to the PCT increase, including viruses, such as influenza, SARS-CoV-2, and invasive candida, and altered renal function, which is a common feature of critical ill patients in the intensive care unit [[17], [18], [19]]. Beyond the impact that the inappropriate order of a biomarker could have on patient management, the economic aspect must also be considered. Indeed, PCT is an expensive test [20]. Thus, the appropriate use of PCT is strongly sought-after.

According to Smellie, a strategy to improve the appropriateness in laboratory medicine is to introduce valuable interventions to decrease the rate of inappropriate test requests, such as applying gating policies and “traffic light” systems, especially for complex and costly tests [21,22].

In this study, we developed several ML models to possible devised a strategy to mitigate the number of inappropriate orders of PCT. Models were built considering PCT positivity (>0.5 ng/mL) as outcome and selecting as features several CBC parameters (including MDW), 1 biochemical parameter (CRP) and 2 demographical parameters (age and sex). We applied a cut-off of 0.50 ng/mL because it is the most used for detecting sepsis in clinical practice.

All features included were selected due to their reduced cost, short turnaround time, and wide availability in all clinical wards; no clinical parameters were instead included.

Among the ML models developed, as expected, RF, SVM, and XGBT showed the best performances in terms of accuracy, kappa, sensitivity, specificity, PPV, NPV, and AUC. Variable importance plots (Fig. 4) suggests that CRP, MDW and PLT were the most contributing variables to the models. With this regard, it is interesting to note that the AUC of the univariates models with only MDW or CRP have an AUC well below the AUC of the ML models. However, despite high accuracy and AUC, sensitivity of the three ML models remained below 0.70, hindering a direct use of these biomarkers as a simple screening tool before PCT order. Nevertheless, a possible use of these ML models in combination with clinical information, i.e. sepsis score and clinical presentation, cannot be excluded. However, this strategy was not evaluated in this study. The low sensitivity found here is someway unexpected, considering that both CRP and MDW are high sensitivity biomarkers. A possible explanation could rely on PCT elevation factors other than sepsis; unfortunately, clinical history of these patients was available only in a minority of cases, preventing the recognition of sepsis-specific PCT elevations.

Accordingly, although the aim of the study was to define an alternative strategy for PCT ordering, not being restricted to any specific clinical setting or patient group, we recognize that the weak or wide selection criteria applied in this study could have introduced a bias, which may reduce the robustness of our results.

A further possible explanation may be found in the cut-off for PCT positivity selected for this study: although the 0.5 ng/mL level if frequently applied in many settings for detecting sepsis, we cannot exclude that the applied PCT threshold may have played a role in the low sensitivity obtained here.

Specificity peaked at 0.90–0.91 for some ML models, suggesting that, for patients predicted as positives by the ML models, particularly with high pre-test probability due to clinical presentation, a PCT order could be avoided. However, this observation remains a speculative result, given in our design the lack of a clinical outcome which is needed to confirm the applicability of our models.

In addition to what has been already pointed out above (no clinical information included, no sepsis definitive diagnosis), another limitations of this study are the modest performance of all MLs developed and the lack of external validation. However, the specific study design applied (train:validation:test design, with internal validation) we believe may in part mitigate this limit.

### Ethics statement

The study protocol was approved by the Ethics Committee of the University Hospital of Palermo (nr 07/2019) and was performed in accordance with the current revision of the Helsinki Declaration. Informed consent was waived because we retrospectively recorded anonymous data.

## Data availability statement

Data will be made available on request.

## CRediT authorship contribution statement

**Luisa Agnello:** Writing – original draft, Conceptualization. **Matteo Vidali:** Writing – original draft, Software, Investigation, Formal analysis. **Anna Maria Ciaccio:** Writing – review & editing, Writing – original draft. **Bruna Lo Sasso:** Writing – review & editing. **Alessandro Iacona:** Formal analysis, Data curation. **Giuseppe Biundo:** Data curation. **Concetta Scazzone:** Writing – review & editing. **Caterina Maria Gambino:** Writing – review & editing, Data curation. **Marcello Ciaccio:** Writing – review & editing, Visualization, Validation, Supervision, Conceptualization.

## Declaration of competing interest

The authors declare that they have no known competing financial interests or personal relationships that could have appeared to influence the work reported in this paper.
